# What Is a Poison?

**Published:** 1896-04

**Authors:** 


### What is a Poison?

This question is propounded to the editor of *The National Druggist* by a correspondent who criticizes the definition of the word "poison" as given by many of the dictionaries. Says this correspondent:

"Webster says a poison is 'any agent which, when introduced into the animal organism, is capable of producing a morbid, noxious, or deadly effect.' Now, should there not be a limitation as regards quantity of the substance? It seems so to me; because there is scarcely a substance known which, if taken too freely, will not produce morbid, noxious, and even deadly effects."

To this query *The Druggist* replies editorially as follows:

"Your criticism of the definition given by Webster is entirely justifiable. The definition of the word given in Dunglison's Medical Dictionary is almost identical with that of Webster, and so is that of Dr. Billings in his great National Medical Dictionary. An English authority, whose name escapes us, defines a poison as 'a drug that kills rapidly when administered in small quantity,' which, while it gives the element missing (the limitation referred to by the querist), is far more liable to criticism than those quoted. All poisons are by no means drugs—as witness the poison of typhus, of malaria, etc. A celebrated English toxicologist, recently deceased, we believe, Dr. Melmott Tidey, defined a poison as 'any substance which, otherwise than by the agency of heat or electricity, is capable of destroying life by chemical action or its physiological effects upon the system.' This, too, is not entirely satisfactory, as admitted by the author, who confessed the difficulty of giving a true and comprehensive definition. If it were true, there is scarcely a substance in the whole armamentarium of medicine that would not fall under the term. Nobody, for instance, thinks or speaks of quinine as a poison, and yet there are numerous instances recorded wherein it has caused death, to say nothing of the 'morbid' and 'noxious' effects of which we have ample evidence every day. Glycerine, too, merely a feeble laxative when taken into the stomach through the mouth, when introduced into the 'animal organism' by direct infection into the blood causes extreme nervous perturbation, and, in the lower animals, death.

"It would seem to us, therefore, that the following definition would be more nearly correct and comprehensive:

"Any substance which, if introduced into a living organism in small amount, or quantities beyond and over a certain definite limit, which latter is variable in each substance and for each class of organism, is capable of destroying life, either by chemical action or by its physiological effects. Like Dr. Tidey, we believe that 'if a substance is a poison it is deadly—if it is not deadly it is not a poison.' Substances which do not kill are merely noxious or hurtful."

